# The Brazilian Longitudinal Study of Adult Health (ELSA-Brasil): the best science is providing health for all

**DOI:** 10.1590/1516-3180.2018.1366191118

**Published:** 2018-12-13

**Authors:** Paulo Andrade Lotufo

**Affiliations:** I MD, DrPH. Full Professor, Department of Internal Medicine, Faculdade de Medicina da Universidade de São Paulo (FMUSP), São Paulo (SP), Brazil.

*Two situations: March 2018. Duke University, United States. The head of the United States National Health Institutes opens a summit conference of world epidemiology, by presenting the 60 most essential cohort studies currently in existence. Among the first slides, there is a world map with the area corresponding to Brazil, showing the presence in this country with a cohort study. It is the Brazilian Longitudinal Study of Adult Health (ELSA-Brasil), a research project that originated in and was financed in this country that has met all the quality requirements to be in the first team of world science. August 2004. Auditorium of Hospital Universitário, University of São Paulo (USP), Brazil. Researchers from USP, FIOCRUZ (Rio de Janeiro) and the Federal Universities of Minas Gerais, Rio Grande do Sul, Espírito Santo and Bahia launch a proposal to study the determinants of heart disease and diabetes in a Brazilian population, after two days of meetings. The plan of what would become ELSA-Brasil was born.*

Between these two times, many things have happened. In 2005, the Brazilian Ministry of Health incorporated the proposal that had been drawn up in August 2004 and, together with the Ministry of Science and Technology, issued a call for financing a multicenter study on cardiovascular diseases and noncommunicable diseases. This public call was won by the six universities that began to organize ELSA-Brasil.

In 2006, 2007 and the first half of 2008, questionnaires, manuals and research protocols were created. Facilities for the clinical investigation were either built or renovated in the six universities. An unprecedented cryobiology center was established to serve the entire study. In parallel, ultrasound and electrocardiogram reading centers started to work.

Thus, in August 2008, everything was ready for the first major epidemiological project in Brazil. The main thing that was still missing was the ELSA participants. When the invitations to participate were issued, there was an immediate and immense response in the six universities. The number of participants was so large that the organizers were forced to stop admitting participants when the total reached 15,105 because of the limit on resources. The initial contact at the workplace, followed by the extended battery of examinations, was very well received.

After ten years, the main players in this project, i.e. the ELSA-Brasil subjects, were still all actively participating through responding to telephone contacts year after year. A second wave of visits was organized in 2012-14 and a third wave of visits in 2017-18. So far, more than 200 original articles using data from ELSA-Brasil have been published; 100 theses and dissertations have been defended; more than 1,000 master’s and doctoral students and clinical research fellows have been trained and then incorporated into the labor market; and Brazilian epidemiology has been integrated with the main scientific centers worldwide.

The impact of ELSA-Brasil cannot be measured solely in terms of its publications. Brazilian studies have begun to adopt the research methods of this study and the cryobiology model. Clinical laboratories have adopted standards that were obtained through ELSA-Brasil, rather than using those coming from foreign populations.

During this time, intensive collaborations have been established with universities overseas, such as Harvard University (Boston, USA), Brown University (Providence, USA), Johns Hopkins University (Baltimore, USA), University of Miami (Miami, USA), University of North Caroline (Chapel Hill, USA), University of Wisconsin (Madison, USA), Erasmus University (Rotterdam, Netherlands) and University of Amsterdam (Amsterdam, Netherlands).

However, the most important outcome has been the production of guidelines for application in the Brazilian National Health System relating to diet, hypertension, diabetes, renal disorders, mental disorders, cognitive dysfunction, headache and thyroid diseases. It completes a virtuous cycle: funding from the Ministry of Health for science has produced returns that benefit all citizens through a new approach towards non-communicable diseases.

The trajectory of ELSA-Brasil shows that it is possible for Brazilian scientists to work together with common goals over the long term, while revealing the high proportion of selfless people who are willing to volunteer with actions that will benefit everyone for a long time.

Going back to what happened at the summit meeting of world epidemiology in the United States in March 2018: Brazil does not have a nuclear arsenal, but it is armed with science to improve the living conditions of humanity, thereby contributing towards understanding the relationship between health and disease.
